# Diversity, Equity, and Inclusion in the Melanoma Research Community

**DOI:** 10.1111/pcmr.13087

**Published:** 2023-04-24

**Authors:** Marie E. Portuallo, David Y. Lu, Gretchen M. Alicea, Joel Bolling, Rebecca Lee, Jennifer McQuade, Allison Betof Warner, Michael Davies, Ashani Weeraratna, Jessie Villanueva, Vito W. Rebecca

**Affiliations:** 1 Johns Hopkins University; 2 University of Manchester; 3 The Wistar Institute; 4 MD Anderson Cancer Center; 5 Memorial Sloan Kettering Cancer Center; 6 Cornell Unviersity

## Abstract

The inaugural Diversity and Inclusion in Science Session was held during the 2021 Society for Melanoma Research (SMR) congress. The goal of the session was to discuss diversity, equity, and inclusion in the melanoma research community and strategies to promote the advancement of underrepresented melanoma researchers. An international survey was conducted to assess the diversity, equity, and inclusion (DEI) climate among researchers and clinicians within the Society for Melanoma Research (SMR). The findings suggest there are feelings and experiences of inequity, bias, and harassment within the melanoma community that correlate with one’s gender, ethnic/racial group, and/or geographic location. Notably, significant reports of inequity in opportunity, discrimination, and sexual harassment demonstrate there is much work remaining to ensure all scientists in our community experience an academic workplace culture built on mutual respect, fair access, inclusion, and equitable opportunity.

## Introduction

“Not everything that is faced can be changed, but nothing can be changed until it is faced”-James Baldwin

Structural oppression, an inequitable atmosphere, biased practices, and lack of inclusivity have historically plagued arenas of higher education and the academic community, with plentiful evidence that these issues continue to persist. To better understand the experiences with and perceptions of diversity, equity, and inclusion (DEI) within the melanoma researcher and clinician community, the Society for Melanoma Research (SMR) performed a diversity, equity, and inclusion (DEI) climate survey in the Summer of 2021. Results from this survey will provide insights into ongoing challenges faced by members the SMR community and help to focus future DEI efforts to truly foster a diverse, equitable, and inclusive SMR community.

## Methods

### Participants

The Society for Melanoma Research (SMR) climate survey was distributed to more than 5,000 members of the global SMR community, receiving 118 responses. Participants were asked about their gender, ethnic/racial group, sexual orientation, and continent of current residence with the option to answer these questions using their own terminology. Participants largely responded as White, Black, Asian, or Hispanic/Latino/a for their racial/ethnic group, male or female for their gender, and heterosexual or homosexual for their sexual orientation. Out of the 118 respondents, 41.5% reported their current sex as male, 57.6% reported as female, and 0.9% preferred not to answer this question. In regard to gender identity, 56.8% reported themselves as woman, 39.8% as man, 0.8% as genderqueer/gender non-conforming, and 2.5% preferred not to answer. The continent of participants’ current residence was divided into North America, Europe, Australia, and Other (answers from South America, Africa and Asia were pooled due to low number of respondents to allow for statistical analyses) after all responses were collected. Where denoted, N refers to the total number of participants from that particular group that responded to that question (Table 1).

### Statistical Methods

Frequency tables were created to summarize survey responses for each question, and Fisher’s exact test was performed to determine nonrandom associations between groups and responses.

## Results

### Inequity in Opportunities

Respondents were asked to attest whether they felt inequity for opportunities in their local lab/clinic or at the institutional/university level. In regard to ethnic/racial group, 44.8% of non-white respondents (N=29) felt they missed out on opportunities in their lab/clinic due to their race/ethnicity relative to among only 8.4% of their white counterparts (N=83) (P value < 0.001) (Table 1). Additionally, 39.3% of non-white respondents (N=28) reported feeling a lack of opportunity in the lab/clinic due to their gender, regardless of gender; whereas only 18.1% of their white counterparts (N=83) reported the same feeling (P value = 0.037).

These reports of inequity were also consistent at the departmental/institutional level. 34.5% of non-white respondents (N=29) reported feeling a lack of opportunity in the department/institution due to their race/ethnicity versus 12.2% of white respondents (N=82) (P value = 0.011) (Table 1). In regard to geographical region, 34.3% of North American (N=70) versus 66.7% of Australian respondents (N=15) reported feeling a lack of opportunity in the department/institution due to their gender (P value = 0.039). Notably, overall 40% of female respondents (N=65) felt a lack of opportunity in their department/institution due to their gender.

### Discrimination

When asked about experiencing discrimination, 31% of non-white respondents (N=29) versus 10.8% of white respondents (N=83) reported experiencing discrimination within their lab and/or clinic (P value = 0.018). No significant difference for experiencing discrimination was found at the institutional level between non-white and white respondents, suggesting environments of discrimination experienced by non-white scientists may be most pronounced at the local lab/clinic level.

When questioned about witnessing discrimination in the lab/clinic, there was no statistically significant difference between ethnic/racial groups. 26.1% of respondents (N=111) witnessed discrimination in their local lab/clinic; 33.3% of white respondents (N=81) and 35.7% of non-white respondents (N=28) reported witnessing discrimination at their department/institution (Table 1).

In regard to gender, 36.4% of female respondents (N=66) versus 18.4% of male respondents (N=49) reported *feeling* discriminated against at their respective department/institution (P value = 0.03). In concordance with these findings, 43.3% of female respondents (N=67) versus 19.6% of male respondents (N=46) reported *witnessing* discrimination in the department/institution (P value = 0.009). No statistically significant results regarding an association between gender and feelings of discrimination were reported in the lab/clinic.

In regards to continent of origin, 37.1% of North American (N=70) and 57.1% of Australian respondents (N=14) reported witnessing discrimination in their department/institution, while only 5.3% of European respondents (N=19) shared these experiences (P value = 0.005) (Table 1). When categorizing responses to discrimination based on rank/position, the data indicates that 60% of junior faculty (N=20) reported feeling discriminated against in the department/institution in comparison to 26.9% of senior faculty/leadership (N=52) (P value = 0.003). Among all participants surveyed, 28.9% reported feeling discriminated against in their department/institution.

Overall, there was an elevated number of females in junior faculty than senior faculty positions among the respondents (not statistically significant). Among both senior and junior faculty, the percentage of females who reported feeling discriminated is about twice as much as males. When analyzed for each gender, the percentage who feel discriminated is twice as high in junior ranks compared to senior ranks (i.e., twice as many junior males feel discriminated vs senior males, twice as many junior females feel discriminated relative to senior females). Regardless of gender, junior faculty have a high proportion of feeling discriminated.

### Sexual Harassment

The topic of sexual harassment in academia has been recently pushed into the spotlight, with institutions taking action against individuals who abuse their power/position. We asked scientists in the melanoma community whether they experienced and/or witnessed sexual harassment in their lab/clinic or at their institution. Notably, 13.4% of female respondents (N=67), relative to no male respondents (N=48), reported *experiencing* sexual harassment in the department/institution (P value = 0.01) (Table 1). 12.9% and 27.8% of respondents have witnessed sexual harassment occurring in their lab/clinic (N=116) or department/institute (N=115), respectively. If we look at this regarding gender, 30.3% of women (N=66) reported witnessing sexual harassment in the department/institution.

### Fairness in Promotions, Funding, and Raises

A total of 51% of respondents (N=98) reported feeling that promotions, funding, raises, and other opportunities are unfair in *their institution*, with 20 individuals not responding or preferring not to answer (Table 1). A large proportion of those surveyed believe that promotions, funding, raises, and other opportunities are not awarded fairly across the scientific community. Specifically according to gender, 89.1% of female (N=64) versus 72.1% of male respondents (N=43) reported feeling these notions (P value = 0.04); 82.2% of total respondents (N=107) expressed concerns of unfair promotions, funding, raises, and other opportunities across the scientific community. This sentiment appeared to fluctuate across continent of origin; 89.4% of North American (N=66), 85.7% of Australian (N=14), 61.1% of European respondents (N=18), and 1 of 3 respondents from other origins reported feeling promotions, funding, raises, and other opportunities were awarded unfairly across the scientific community (P value = 0.006) (Table 1). It is worth noting that 17% of those surveyed preferred not to respond to the survey question regarding fairness in their institution. 54.2% of North American (N=59) and 50% of European respondents (N=16) reported feeling that promotions, funding, raises, and other opportunities are unfair in *their institution*.

## Discussion and Next Steps

Through our inaugural DEI Climate Survey, we have now begun to face the remnants of structural racism, inequity, and lack of inclusivity that persist in our melanoma research community. As a community, we need to develop best practices to address these concerns to protect the right to a diverse, equitable, and inclusive community each member of our community deserves. Our findings indicate that there are statistically significant differences in feelings and experiences regarding DEI amongst the SMR community. These results further reinforce the need for us to address ongoing oppression, bias, and exclusion faced by many SMR community members. We hope that this complex issue can be addressed by establishing a foundation of actionable recommendations to improve the SMR climate, making it more divers, inclusive, and equitable with the support of leaders of our society. Recommendations we include here for future SMR conference sessions and the future of our community are as follows:

- Highlight the work of scientists from underrepresented groups as a regular feature. This began during the first DEI session in the 2021 SMR meeting and continued during the 2022 SMR meeting in the Global Perspectives session.
- Continue to support and promote women and individuals from underrepresented backgrounds into leadership positions.
- Host SMR meetings in countries/regions outside of the US, Europe, and Australia every 5 or 10 years
- Create a multi-level mentorship program, where early and mid-career scientists from underrepresented groups are given access to resources, professional development opportunities, and increased involvement in SMR activities
- Provide a CV review service and grant writing workshop for people of underrepresented minority backgrounds in the sciences.
- Make recommendations to funding agencies/foundations to make funding processes more equitable.
  – Make reviewer pools more diverse to ensure funding decisions are equitable and free of bias.
  – Encourange funding agencies to develop novel funding mechanisms to support underrepresented scientists that provide financial support for their research, mentoring, and leadership development.
  – Request that funded institutions provide information about their commitment to DEI and plans to enhance diversity and inclusion.
- Be more conscientious of social biases when presenting speakers at conferences or seminars. For example, a study published by ASCO identified that 17% of females were being introduced by first name compared to 3% of males [1]. Recommend that organizing committees intentionally focus on gender, equity, and diversity among speakers, ensuring their visibility.
- Formal reporting mechanism for trainees/scientists to flag discrimination, harassment, etc.

With continued help from the community, we hope to make the professional work environment more diverse, equitable, and inclusive to ensure all SMR members feel welcomed, recognized, and safe.

Our findings/conclusions and feelings of inequality and discrimination are not unique to the melanoma research community. This survey is fairly consistent with previous publications – other professional societies have published on gender, and ethnicity/race within their own communities. For example, EMSO (European Society for Medical Oncology) reported that women constitute less than 15% of leadership roles in their society, and only 30% of committee members overall [2]. In the melanoma community as of 2023, 20% (2 out of 8 total) of the CMR Presidents have been women, with the first woman serving in 2019. Wellcome, a UK-based charitable foundation that supports scientific research in climate change, mental health, and infectious disease, reported a mean ethnicity pay gap of 9.4% compared to white employees [3]. To what extent these pay gaps exist within the melanoma community remain unclear, but will be inquired in the next iteration of this survey. The Academy of Nutrition and Dietetics (AND) showed “underrepresentation of black (2.6%), Asian (3.9%), Latinx (3.1%), Native Hawaiian or Pacific Islander (1.3%), or indigenous (American Indian or Alaskan Native) (0.3%) individuals compared with the US population” [4]. In agreement, a study published by the AACR showed that only 2.3% of oncologists are Black and 5.8% are Latinx, compared to 13% and 18% of the Black and Latinx US populations, respectively. This is further exacerbated by the fact that only 11% of medical students are underrepresented minorities in the sciences [5].

Of note, the sample sizes relating to sexual orientation were too small to be representative of the SMR community; hence, the results were not included in the analysis. Because the number of participants produces a limited study, we will encourage increased future participation from the melanoma research community through physical mailing, electronic mail, and event awareness. Our initial goal was to alert the community of these biases to facilitate dialogue and change, with the future goal of encouraging more participants to be involved in the survey. Although the survey was directed specifically towards those in the SMR community, the melanoma community appears to be a mirror of scientific professions in general. Melanoma research trial participants would always be weighted towards a white population due to its risk factors, but the people that work in the laboratories and clinics do not necessarily follow that same bias. At the next annual SMR meeting, we will poll SMR members for race/ethnicity upon registration to address this question.

We hope that with more survey responders in the next iteration that we would be able to more precisely dissect associations with discrimination, sexual harassment, and opportunity inequity. Overall, there appears to be an agreement among members of the SMR community regarding concerns to fair access and opportunity based on gender, ethnic/racial group, and geographic location. We are confident that the data we will continue to gather will help us better understand the perspectives of our community, prioritize goals and activities, and track our progress. Achieving diversity and inclusion in our society is an ongoing process that requires the commitment and efforts of all SMR members and support of our leaders.

## Figures and Tables

**Table 1:** Breakdown of statistical analyses performed on survey results.

*Inequity in Opportunities*							
**Q1.1**	**Feel a lack of opportunity in the lab/clinic due to gender**						
Group	Yes	No	Total	P			
White	15 [18.1%]	68 [81.9%]	83	0.03687			
Non White	11 [39.3%]	17 [60.7%]	28				
Total	26 [23.4%]	85 [77.6%]	111				
**Q5.1**	**Feel a lack of opportunity in the lab/clinic due to race/ethnicity**						
Group	Yes	No	Total	P			
White	7 [8.4%]	76 [91.6%]	83	< 0.001			
Non White	13 [44.8%]	16 [55.2%]	29				
Total	20 [17.9%]	92 [82.1%]	112				
**Q5.2**	**Feel a lack of opportunity in the department/institution due to race/ethnicity**						
Group	Yes	No	Total	P			
Non White	10 [34.5%]	19 [65.5%]	29	0.01144			
White	10 [12.2%]	72 [87.8%]	82				
Total	20 [18%]	91 [82%]	111				
**Q4.2**	**Feel a lack of opportunity in the department/institution due to gender**						
Group	Yes	No	Total	P			
North America	24 [34.3%]	46 [65.7%]	70	0.03929			
Australia	10 [66.7%]	5 [33.3%]	15				
Total	34 [40%]	51 [60%]	85				
**Q3.2**	**Feel a lack of opportunity in the department/institution due to gender**						
Group	Yes	No	Total	P			
Female	26 [40%]	39 [60%]	65	0.6966			
Male	17 [34.7%]	32 [65.3%]	49				
Total	43 [37.7%]	71 [62.3%]	114				
***Discrimination***					*** ***	*** ***	*** ***
**Q9.1**	**Feel discrimination in the lab/clinic**						
Group	Yes	No	Total	P			
Non White	9 [31%]	20 [69%]	29	0.01761			
White	9 [10.8%]	74 [89.2%]	83				
Total	18 [16.1%]	94 [83.9%]	112				
**Q9.2**	**Feel discrimination in the department/institution**						
Group	Yes	No	Total	P			
Non White	10 [34.5%]	19 [65.5%]	29	0.4712			
White	21 [25.9%]	60 [74.1%]	81				
Total	31 [28.2%]	79 [71.8%]	110				
**Q10.1**	**Witnessed discrimination in the lab/clinic**						
Group	Yes	No	Total	P			
Non White	9 [31%]	20 [69%]	29	0.4731			
White	20 [24.4%]	62 [75.6%]	82				
Total	29 [26.1%]	82 [73.9%]	111				
**Q10.2**	**Witnessed discrimination in the department/institution**						
Group	Yes	No	Total	P			
Non White	10 [35.7%]	18 [64.3%]	28	0.8207			
White	27 [33.3%]	54 [66.7%]	81				
Total	37 [33.9%]	72 [66.1%]	109				
**Q9.3**	**Feel discrimination in the lab/clinic**						
Group	Yes	No	Total	P			
Female	13 [19.4%]	54 [80.6%]	67	0.4468			
Male	6 [12.2%]	43 [87.8%]	49				
Total	19 [16.4%]	97 [83.6%]	116				
**Q9.4**	**Feel discrimination in the department/institution**						
Group	Yes	No	Total	P			
Female	24 [36.4%]	42 [63.6%]	66	0.03921			
Male	9 [18.4%]	40 [81.6%]	49				
Total	33 [28.7%]	82 [71.3%]	115				
**Q10.4**	**Witnessed discrimination in the department/institution**						
Group	Yes	No	Total	P			
Female	29 [43.3%]	38 [56.7%]	67	0.009256			
Male	9 [19.6%]	37 [80.4%]	46				
Total	38 [33.6%]	75 [66.4%]	113				
**Q10.8**	**Witnessed discrimination in the department/institution**						
Group	Yes	No	Total	P			
North America	26 [37.1%]	44 [62.9%]	70	0.005221			
Europe	1 [5.3%]	18 [94.7%]	19				
Australia	8 [57.1%]	6 [42.9%]	14				
Other	1 [33.3%]	2 [66.7%]	3				
Total	36 [34%]	70 [66%]	106				
**E1**	**Feel discriminated against in the department/institution**						
Group	Yes	No	Total	P			
Senior Faculty/Leadership	14 [26.9%]	38 [73.1%]	52	0.002977			
Junior Faculty	12 [60%]	8 [40%]	20				
Trainee/Other	7 [16.7%]	35 [83.3%]	42				
Total	33 [28.9%]	81 [71.1%]	114				
***Sexual Harassment***					*** ***	*** ***	*** ***
**Q11.4**	**Experienced sexual harassment in the department/institution**						
Group	Yes	No	Total	P			
Female	9 [13.4%]	58 [86.6%]	67	0.009914			
Male	0 [0%]	48 [100%]	48				
Total	9 [7.8%]	106 [92.2%]	115				
**Q12.1**	**Witnessed sexual harassment in the lab/clinic**						
Group	Yes	No	Total	P			
Non White	3 [10.3%]	26 [89.7%]	29	0.7551			
White	12 [14.8%]	69 [85.2%]	81				
Total	15 [13.6%]	95 [86.4%]	110				
**Q12.2**	**Witnessed sexual harassment in the department/institution**						
Group	Yes	No	Total	P			
Non White	7 [24.1%]	22 [75.9%]	29	0.6359			
White	24 [30%]	56 [70%]	80				
Total	31 [28.4%]	78 [71.6%]	109				
**Q12.4**	**Witnessed sexual harassment in the department/institution**						
Group	Yes	No	Total	P			
Female	20 [30.3%]	46 [69.7%]	66	0.4036			
Male	11 [22.9%]	37 [77.1%]	48				
Total	31 [27.2%]	83 [72.8%]	114				
	**Witnessed sexual harassment in the lab/clinic (overall)**						
	Yes	No	Total				
	15 [12.9%]	101 [87.1%]	116				
	**Witnessed sexual harassment in the department/institution (overall)**						
	Yes	No	Total				
	32 [27.8%]	83 [72.2%]	115				
***Fairness in Promotions, Funding, Raises, Etc***					*** ***	*** ***	*** ***
**Q13.3**	**Promotions, funding, raises, and other opportunities are awarded fairly across the scientific community**						
Group	Yes	No	Total	P			
Female	7 [10.9%]	57 [89.1%]	64	0.03756			
Male	12 [27.9%]	31 [72.1%]	43				
Total	19 [17.8%]	88 [82.2%]	107				
**Q13.7**	**Promotions, funding, raises, and other opportunities are awarded fairly across the scientific community**						
Group	Yes	No	Total	P			
North America	7 [10.6%]	59 [89.4%]	66	0.005677			
Europe	7 [38.9%]	11 [61.1%]	18				
Australia	2 [14.3%]	12 [85.7%]	14				
Other	2 [66.7%]	1 [33.3%]	3				
Total	18 [17.8%]	83 [82.2%]	101				
**Q13.8**	**Promotions, funding, raises, and other opportunities are awarded fairly in your institution**						
Group	Yes	No	Total	P			
North America	27 [45.8%]	32 [54.2%]	59	0.8912			
Europe	8 [50%]	8 [50%]	16				
Australia	6 [42.9%]	8 [57.1%]	14				
Other	2 [66.7%]	1 [33.3%]	3				
Total	43 [46.7%]	49 [53.3%]	92				
	**Follow up Analysis Regarding Junior vs Senior Faculty Diversity**				**Senior Faculty/Leadership**	**Junior Faculty**	**Trainee/Other**
					Full Professor	Assistant Professor	Housestaff Physician (Fellow)
	**Junior**	**Senior**	**Total**		Associate Professor		Graduate Student
**White**	15 [71.4%]	40 [76.9%]	55	P = 0.359	Attending physician		Postdoc
**Non White**	4 [19.0%]	11 [21.2%]	15		Dr		Post Baccalaureate Fellow
**Prefer Not to Answer**	2 [9.5%]	1 [1.9%]	3		Chief Scientific Officer		Staff Scientist
**Total**	21	52	73		Senior Director, Scientific Program		Research Nurse
					senior physician		Technician/Research Assistant
	**Junior**	**Senior**	**Total**		MEDICAL DIRECTOR		
**Male**	8 [38.1%]	27 [51.9%]	35	P = 0.213			
**Female**	12 [57.4%]	25 [48.1%]	37				
**Prefer Not to Answer**	1 [4.8%]	0 [0 %]	1				
**Total**	21	52	73				

Question: Is there a correlation among respondants between more junior faculty being a more diverse group (typically) than more senior faculty? If so, that observation could be worth mentioning.

There are indeed more females among junior faculty than senior faulty (but this difference is not statistically significant), Also, among both senior and junior faculty, the % of females who feel discriminated is about twice and much as males. But for each gender, the % who feel discriminated is twice as high in junior compared to senior (twice as many junior male feel discriminated vs senior male, twice as many junior female feel discriminated as senior female). So regardless of gender, junior faculty have a high proportion of feeling discriminated. (ie gender is not the reason why we see differences between junior and senior faculty)
